# The Surgical Treatment of Stone

**Published:** 1912-01-20

**Authors:** John Ward Cousins

**Affiliations:** Consulting Surgeon to the Royal Portsmouth Hospital.


### THE SURGICAL TREATMENT OF STONE.

### A Review of its Practice during the Last Sixty Years.—I.

By JOHN WARD COUSINS, M.D., F.R.C.S., Consulting Surgeon to the Royal Portsmouth Hospital.

The present position of any branch of practical science very rightly tends to overshadow all the early stages of its evolution, and progress may have been both rapid and brilliant. A glance backwards, however, is always full of interest to earnest students, and it may even do something to promote increased activity and new efforts.

### The Unqualified Operator.

In bygone days surgical procedures were considered quite outside the province of regular physicians, who undertook the treatment of diseases by empirical remedies, and almost all operative treatment was carried on by unqualified practitioners. For many centuries the rough manipulations for the removal of stone in the bladder—styled cutting on the grip—underwent very little modification. No regular efforts were made to diagnose the disorder, and no form of sounding through the urethra was practised; but one or two fingers of the left hand were passed into the rectum, and the right hand firmly pressed over the vesical region and the stone pressed from behind forwards against the neck of the bladder and perineum, and then an incision was made above the anus directly down to the stone. Of course, such an operation must have been attended with many dangers, and we may be certain that in those early times it was followed by a very high mortality.

In the early part of the seventeenth century the Marian operation was introduced into France, and soon superseded the older methods. A guide was passed down the urethra and the bladder opened by a deep lateral incision, which was regulated by the size of the stone. During the eighteenth century many eminent French surgeons performed lithotomy by dilatation of the wound, and then after many years this modification of the operation was practised with a gorget, which was passed along a grooved staff into the bladder. After many more years the gorget fell into disuse, and the knife was recognised by nearly all surgeons as the best instrument for opening up the deeper part of the perineal wound.

It is not my intention to enter further into the history of these ancient methods. The early treatment of vesical stone in this country will be for ever associated with the name of Cheselden, the distinguished surgeon of St. Thomas's Hospital in the early part of the eighteenth century. He greatly improved by his earnest labours the lateral operation, and rendered its performance much easier and more safe in days when all surgical operations were attended with acute suffering.

### My Early Opportunities.

I shall endeavour to record my own recollections on the progress which has taken place in the treatment of stone in the bladder during the last sixty years. In the early part of my professional life I had many opportunities of witnessing cases of lithotomy at the Royal Portsmouth Hospital and afterwards in London at St. Thomas's Hospital. During the period in which I acted as house surgeon lateral lithotomy and occasionally bilateral were the operations generally practised, and my first experiences deeply impressed me with the fact that these operations were always attended with great risk in patients suffering from chronic organic disease and prolonged constitutional disturbance. At that time many serious complications were explained as the results of too free division of the urethral structures, and imperfect slopping of the wound from the prostate downwards, which induced serious inflammation of the vesical walls and dangerous urinary infiltration; but it has now long been my opinion that in the majority of these cases these complications arose from the septic condition of the urinary organs and the absence of satisfactory surgical precautions.

### Student Days.

The instruments employed in these early days for the performance of lithotomy were marked by many variations. During my student days in the early fifties I assisted at many operations very skilfully performed by my old friend the late Dr. E. J. Scott, who enjoyed a great surgical reputation at Portsmouth. In the operation for stone he always employed a concealed knife fitted with a screw arrangement, by which the extent of the cutting edge could be regulated. After opening the urethra he slipped the slender sheath into the groove of the staff, and, as soon as the point entered the bladder, the cutting edge was projected and the deep incision completed in withdrawing the instrument. At St. Thomas's Hospital some of the surgeons generally used a broad and pointed knife for making the first part of the incision, and then they exchanged this for a blunt instrument having something of the shape of the old-fashioned gorget. The majority of the operations were performed by completing the whole incision with a long, broad-bladed knife curved slightly backwards at the point. My early experience led me to the conclusion that a pointed knife was the safest and best instrument in all cases of lateral lithotomy.

In the year 1860 I commenced my surgical practice at Portsmouth, and at that period cases of vesical stone were of frequent occurrence in the neighbourhood and surrounding country.

### Lateral Operation Universally Practised Then.

The lateral method was then the operation almost universally practised, and it had the reputation of being one of the most successful operations in surgical science, although the technical details were mingled with many debatable questions chiefly arising in connection with the deep and delicate manipulations. During the performance of the operation the eye could not completely direct the hand as it can do when the structures to be divided are in a region exposed to view. The surgeon had to rely upon the sensibility of the hand and the sense of touch. His chief instrument was the left forefinger, and his main object was to reach the staff at the right point, and then, directed by his finger, to cling to it as his guide into the bladder.

Much has been written in the past upon the method of performing lateral lithotomy, but any reference to these interesting records would be beyond the scope of my paper, which is intended to be only a retrospect of my own experience.

### Surgeons' Idiosyncrasies.

Now nearly every operating surgeon that I have known—and I have had the pleasure and privilege of meeting many—has been the possessor of some little manipulative manœuvre or of some special form of scalpel or forceps which have proved of great assistance in the performance of this operation. Some years ago one of my colleagues strongly pressed upon me the importance of having the staff firmly fixed in the upright position and well hooked up under the pubic arch. My old friend the late Mr. Reginald Harrison objected to the notion of holding the guide with the intention of making it prominent in the perineum. The late Mr. Cadge, of Norwich, told me that the handle of the staff should be made to incline towards the abdomen in making the first incision for the purpose of bringing the urethra as near as possible to the surface. I have seen a very distinguished surgeon use a large and broad knife in lateral lithotomy, and another loudly expatiate on the value of the old-fashioned gorget. No doubt in the hands of an experienced operator the bistouri caché was a safe instrument to use in making the deep part of the incision, and some years ago it was regarded with special favour by many Continental surgeons. On one occasion I was present at an operation when the concealed knife was actually fractured in the groove of the staff, but the surgeon took the accident very coolly, and with very little delay he successfully removed three large stones from the bladder.

### Lateral Lithotomy in Children.

Lateral lithotomy in young children under four years of age has never been regarded as easy of performance, for the perineal space is very limited, the urethral structures are very tender and delicate, and the bladder is situated high up behind the pubic arch. At all times in making the deep incision, and in insinuating the finger between the wall of the membranous urethra and the staff, the greatest care was necessary. I have been present in several cases in which the operator unfortunately failed to enter the narrow neck of the bladder, and in the effort to do so the prostate was pushed upwards and the finger entered the space between the rectum and the staff.

Now I candidly admit that I have never done lateral lithotomy on a young child without feeling some anxiety, and this fact led me to give the matter very careful consideration. Thirty-five years at least have now passed away since I suggested a simple alteration in the ordinary guide then in general use, with the hope of rendering this operation more easy and more free from danger. The instrument * was a small modified rectangular staff with a rounded angle and rather a long beak, and the lateral groove which began half an inch above the angle and terminated about the same distance from the point. Some lithotomists at that time did not like a staff with a long beak, and considered it difficult to introduce into the bladder of a child, but I found that this could very easily be done. Moreover, the round and sharp angle was a great assistance in making the deep incision, for as soon as the angle was exposed the point of the left finger informed the operator that he was on the angle, and directly the side of the nail was well insinuated in the groove he could be quite certain that he had reached the beak, and that a simple movement of the knife would open the bladder. The long beak rendered also the introduction of the finger over the staff both easy and safe, as the concave border always proved the best guide to the stone in operations upon children.

### Median Lithotomy.

With reference to median lithotomy, I have very little to record. About the early seventies it received considerable attention over the country, and especially at Norwich. In two simple cases only I performed this operation, but the limited space for the knife with the bulb and artery above and the bowel behind, and especially the difficulty of exploring the interior of the bladder, rendered it a very unsatisfactory proceeding. I must admit, however, I saw several cases in which small stones were successfully removed by this method; but after a few years the disadvantages and risks often appeared far greater than with the lateral operation, and so it fell out of favour, and, like many other performances in this world, it slowly went out of fashion.

### Influence of Antiseptic Precautions.

I must not omit to refer to the great reformation in practice which followed the universal adoption of the Listerian surgery. It is quite true that lithotomy had been for some years performed with increasing success, and that greater cleanliness and better methods of dressing wounds had done much to remove the dangers of surgical treatment, and in a measure to prepare the way for the advent of the aseptic system, which fortunately shook the old foundations and revolutionised the art. During the last quarter of the past century the new system has yielded greatly improved results in vesical operations, as well as in the surgical treatment of every other department. The important surgical precautions have reduced the risks of operation, prevented the development of septic troubles and those complications which formerly arose from the entrance of poisonous products through the wound. I have still a vivid recollection of my early aseptic operations done in a thick cloud of steam and under the protection of a light indiarubber coat.

* *British Medical Journal,* January 29, 1881.

(*To be continued.*)

